# Characterising Lipid Transfer Protein Allergy in UK Adults

**DOI:** 10.1111/cea.70050

**Published:** 2025-03-31

**Authors:** I. J. Skypala, B. Olivieri, G. Scadding

**Affiliations:** ^1^ Royal Brompton & Harefield Hospitals Part of Guys & St Thomas NHS Foundation Trust London UK; ^2^ Department of Allergy & Clinical Immunology Imperial College London UK; ^3^ Allergy Unit University Hospital of Verona Verona Italy

**Keywords:** allergic rhinitis, allergy, co‐factor, lipid transfer protein, pollen

1

**Summary**

- LTP‐allergic UK adults experience moderate–severe symptoms to foods, including those sensitised to pollens/PR‐10 allergens.
- Co‐factors, especially exercise and alcohol, affect nearly 50% of patients.

**Abbreviations**
AAI: adrenaline autoinjector
AD: atopic dermatitis
A&E: accident and emergency
FA: food allergy
FASS: Food Allergy Symptom Severity score
LTP: lipid transfer protein
PFS: pollen food syndrome

To the Editor,

1

Although Pollen Food Syndrome (PFS), involving reactions to pathogenesis‐related proteins, is the most prevalent plant food allergy affecting United Kingdom adults [1], lipid transfer proteins (LTP) are an increasing cause of allergic reactions to plant foods [2, 3, 4]. To evaluate the clinical features of LTP allergy, we undertook an audit of records of adult patients seen at the Royal Brompton & Harefield Hospitals, London, between 2012 and 2022, who had a positive IgE test (≥ 0.35 KUA/l) to the peach LTP, Pru p 3. The anonymised and retrospective nature of the data collection meant no ethical approval was required. Statistical analysis (SPSS.inc. 29.0, Chicago, IL) included differences in quantitative (Student's *t*‐test) and qualitative data (Pearson chi square).

Of 1642 adults tested to Pru p 3, 308 (19%) were positive, 285 of whom had complete data. Of these, 157 (55%) were diagnosed with LTP allergy (LTP+ve) and 128 (LTP‐ve) with an alternative diagnosis on the basis of clinical history and test results (Table 1). There were no differences in age, gender or seasonal allergic rhinitis (SAR), but asthma and atopic dermatitis were more commonly reported by the LTP‐ve patients. Food triggers were similar in both groups, but LTP + ve patients frequently reacted to composite foods (containing products of both plant origin and processed animal origin), tomato puree/soup, dried fruit, peaches and grapes. Urticaria, angioedema, and generalised pruritus were more often reported by the LTP+ve cohort, 59% of whom also had severe Grade 4/5 symptoms compared to 46% of LTP‐ve patients (*p* < 0.001) (FASS‐5 symptom severity score [5]). The LTP+ve group were also more likely to have attended a hospital emergency department and prescribed adrenaline autoinjectors. Reactions linked to co‐factors were reported by 72 (46%) of the LTP+ve group and 13 (10%) of the LTP‐ve group, most often exercise (38%) and/or alcohol (29%), with 21/72 only reacting to foods when a co‐factor was present.

**TABLE 1:** A comparison of the clinical features of patients sensitised to Pru p 3 who have a diagnosis of LTP allergy, compared to those sensitised to Pru p 3 but with an alternative diagnosis.

		LTP‐allergy diagnosis ‘LTP+ve’ (157)	Other diagnosis ‘LTP‐ve’ (128)	*p* value (Pearson chi‐sqare)
Additional or Primary Diagnosis	Pollen Food Syndrome (PFS)	33	36	
	Primary food allergy	13	37	
	PFS + primary food allergy	3	22	
	Unknown	0	33	
Demographic data	Gender (Female)	101 (64%)	72 (56%)	0.165
	Mean age on referral (years)	33.2	33.6	0.792 (*t*.test)
Co‐morbidities reported by ≥ 15 patients	Asthma	52 (33,1%)	73 (57%)	< 0.001
	Seasonal Allergic Rhinitis	111 (71%)	96 (75%)	0.418
	Atopic Dermatitis	48 (31%)	76 (59%)	< 0.001
Food triggers reported by ≥ 15 patients	Any composite food	76 (48%)	33 (26%)	0.0001
	Peanut	49 (31%)	36 (28%)	0.571
	Apple	40 (26%)	30 (23%)	0.691
	Peach	33 (21%)	12 (9%)	0.007
	Fruit other/unspecified	32 (20%)	17 (13%)	0.114
	Nut other/unspecified	28 (18%)	23 (18%)	0.977
	Almond	26 (17%)	13 (10%)	0.118
	Tomato puree/soup	22 (14%)	4 (3%)	0.001
	Veg – other	22 (14%)	16 (13%)	0.709
Symptoms reported by ≥ 15 patients	Generalised Pruritis	52 (33%)	21 (16%)	0.003
	Urticaria	69 (44%)	25 (20%)	< 0.001
	Periorbital angioedema	55 (35%)	17 (13%)	< 0.001
	Angioedema elsewhere	83 (53%)	35 (27%)	< 0.001
	Skin rash	40 (26%)	15 (12%)	0.008
	Sneeze or itch	18 (12%)	5 (4%)	0.037
	Wheeze or cough	36 (23%)	31 (24%)	0.518
	Laryngeal Symptoms	34 (22%)	12 (9%)	0.011
	Shortness of breath	28 (18%)	16 (13%)	0.259
	Itchy mouth/throat	50 (32%)	62 (48%)	0.008
	Lip or tongue swelling	16 (10%)	17 (13%)	0.381
	Abdominal pain	21 (13%)	16 (13%)	0.530
	Vomiting	25 (16%)	17 (13%)	0.452
FASS Symptom severity score	Mild Grade 1	1 (0.6%)	20 (16%)	
	Moderate Grade 2	44 (28%)	42 (34%)	
	Moderate Grade 3	19 (12%)	6 (5%)	
	Severe Grade 4	74 (47%)	49 (39%)	
	Severe Grade 5	18 (12%)	8 (6%)	
Severe reactions	Number of visits to A&E	78 (50%)	28 (22%)	< 0.001
	Used Adrenaline autoinjector	32 (20%)	14 (11%)	0.007
Co‐factor involvement	Total reporting co‐factors	72 (46%)	13 (10%)	< 0.001
	Exercise	59 (38%)	8 (6%)	< 0.001
	Alcohol	45 (29%)	8 (6%)	< 0.001
	Aspirin/NSAID	10 (6%)	1 (1%)	0.015
	Other co‐factor	21 (13%)	5 (4%)	0.006
Pollen sensitisation Positive/total tested (%)	Birch	71/130 (55%)	65/87 (75%)	O.003
	Mixed tree	26/40 (65%)	30/42 (71%)	0.531
	Plane Tree	44/65 (68%)	25/42 (60%)	0.291
	Any tree	101/140 (72%)	85/104 (82%)	0.081
	Grass	97/142 (68%)	101/110 (92%)	< 0.00001
	Tree and/or grass	121/147 (82%)	104/114 (91%)	0.038
	Mugwort	54/99 (54%)	19/59 (32%)	0.006
	Any pollen	123/147 (84%)	105/114 (92%)	0.042

Reported SAR affected over 70% of both groups (*p* = 0.418). Although 82% LTP+ve and 91% LTP‐ve patients had positive tests to tree and/or grass pollen (*p* = 0.038), LTP+ve adults were less often sensitised to Timothy grass ( *Phleum pratense* ) (*p* < 0.00001) and birch ( *Betula verrucosa* ) (*p* = 0.003) and more often sensitised to mugwort ( *Artemisia vulgaris* ) (*p* = 0.006). There was no significant difference in symptom severity score, likelihood of emergency hospital visits or use of AAI between the LTP + ve patients who were/were not sensitised to pollen. The LTP + ve patients who also had a diagnosis of PFS were more likely to have a FASS‐5 score of 4/5 (*p* < 0.0001), attend hospital for a food allergic reaction (*p* = 0.004), experience co‐factor reactions (*p* = 0.0001), report symptoms of urticaria (*p* < 0.0001), angioedema (*p* = 0.02) and reactions to grapes (*p* = 0.01) and tomato puree/soup (*p* = 0.04) when compared to LTP‐ve individuals diagnosed with PFS.

These data help further characterise LTP allergy in a UK population. Apple, peanut, some tree nuts, and other fruit are common UK food triggers, which can make diagnosis difficult as they are also known causes of PFS. However, certain fruits and composite foods were significantly more likely to be reported by the LTP+ve group, which demonstrates the importance of taking a detailed dietary history during the diagnostic pathway. Although symptom type and severity also provide useful clues, eliciting the presence or absence of co‐factors is essential, given the frequency with which they occur in LTP allergy. Exercise and alcohol are major co‐factors in the United Kingdom, unlike Spain and Italy, where non‐steroidal anti‐inflammatory drugs (NSAID) are a more common trigger [6, 7]. The lack of statistical significance between LTP+ve and LTP‐ve groups for NSAIDS as a co‐factor may be due to the low number of cases.

Our data suggests that pollen sensitisation is common in UK individuals who have LTP allergy, and this can complicate diagnosis, especially as the common food triggers of LTP allergy in the UK are very similar to those reported for PFS. However, sensitisation to mugwort appears to be a useful confirmatory marker if LTP allergy is suspected. The high rate of pollen sensitisation might account for 36/157 (23%) LTP+ve patients also having a diagnosis of PFS, some of which was historical, but might reflect the reporting of both mild and severe reactions. It has been reported that individuals with LTP allergy who are co‐sensitised to PR10 and profilins may experience milder reactions [8, 9]. but our data suggests this is not the case in UK adults. Pollen sensitisation might explain why individuals experiencing both mild reactions to raw plant foods and moderate/severe reactions to cooked/processed plant foods were diagnosed with both LTP allergy and PFS. It has been well reported that sensitisation to Pru p 3 is not always diagnostic of LTP allergy, and mild oropharyngeal reactions to raw plant foods alone would almost always lead to a diagnosis of PFS in our population, even if mild sensitisation to LTP were also present.

This study was limited by the retrospective nature of the data collection, which meant that the original assessment and diagnostic methods varied, so not all data was uniform.

In conclusion, we have presented data on a large cohort of UK adults with a diagnosis of LTP allergy. Although there are some similarities in the presentation of LTP allergy in the UK compared to Italy or Spain, there are notable differences, including reactions to composite foods and alcohol being a common co‐factor. Pollen sensitisation complicates diagnosis and may result in a diagnosis of PFS or LTP or both, but our data suggest that co‐sensitisation to PR10 allergens does not result in a milder phenotype of LTP allergy in the UK.

## Author Contributions

I.J.S. collected data, supported the data analysis, wrote and submitted the final manuscript. B.O. collected and analysed the data and designed figures and tables, made comments and approved the final manuscript. G.S. supported and supervised the project, commented on the manuscript and approved the final version for submission.

## Conflicts of Interest

Isabel Skypala has received lecture fees from ThermoFisher, DBV, Touch Independent Medical Education. Bianca Olivieri declares no conflicts of interest. Guy Scadding declares no conflicts of interest.

## Data Availability

The authors have nothing to report.
